# History of a Case of Traumatic Tetanus, Successfully Treated

**Published:** 1820-05

**Authors:** Michele Medici

**Affiliations:** Professor of Physiology in the University of Bologna, First Physician to the Principal Hospital at Bologna.


FOR THE LONDON MEDICAL AND PHYSICAL JOURNAL.

### *History of a Case of Traumatic Tetanus, successfully treated.* By Dr. Michele Medici, Professor of Physiology in the University of Bologna, First Physician to the principal Hospital at Bologna.

A MAN, about twenty-five years of age, of a robust habit, and of a healthy constitution, received, from the bite of a pig, a laceration of the external part of the left hand. Some degree of inflammation came on about the injured parts, but the wound had cicatrized at the end of a few days. Soon after this the patient suffered, without any evident immediate cause, deep-seated pain in the lacerated part, which was in a short time followed by perfect trismus. He was then brought into the hospital. His pulse was frequent and vibrating; there was permanent rigidity of the muscles of the neck, the chest, and the abdomen; the whole of the voluntary muscles were occasionally thrown into violent convulsive action; the head and chest were firmly drawn backwards; the eyes were immovable, had a glistening appearance, and were sunk in their orbits. The skin was burning hot, except on the face and chest, where there were partial sweats. Considering the robust habit and athletic form of the patient, the symptoms of the disease, and that traumatic tetanus is generally of an inflammatory nature, it was resolved that the use of contra-stimulants should be immediately resorted to. Blood-letting, a drachm of cohobated laurel-water* diluted with three ounces of distilled water, and fomentations of the affected hand with oil, constituted the means of cure for several days; but the disease became more and more severe, the tetanic contraction of all the voluntary muscles was carried to the highest degree, and it became almost impossible to introduce any thing into the mouth. The pulse, although it had still some degree of vibration, was yet very low, thread-like, and was easily lost on compression. The face was of a cadaverous hue, and covered with large drops of sweat. At this time the advice of Dr. Comelli, the ordinary physician to some other wards in the same hospital, was requested. Considering, from the lowness and sinking of the pulse, that the medicine might have brought on a diathesis of defect of stimulus, and the blood drawn not having presented a buffy pellicle, at the same time that the patient became worse under the use of sedatives, it was determined that stimulants should be employed. Half-a-drachm of extract of opium, dissolved in a few ounces of wine, was the remedy prescribed; and the above quantity was directed to be administered in the twenty-four hours, provided nothing sinister occurred. The patient had not taken half of the above mixture, when he fell into a state of sopor and muttering delirium: the pulse had become high and vibrating, the heat of the skin was increased, and the contraction of the muscles became still more intense. The assistant physician, Dr. Bacialli, ordered the exhibition of the opium to be suspended; and, on the ensuing morning, it was determined to return with vigour to the use of sedatives. Blood-letting was again employed; two drachms of cohobated laurel-water in four ounces of water, to be taken in the course of the day; tepid baths repeated three times a-day, and continued immersion of the wounded hand in oil, were the remedies now adopted; and, by persevering with the use of them, the patient was at length restored to perfect health.

---

* As this remedy is so commonly resorted to in Italy, and will consequently be frequently mentioned in our histories of cases, we shall give, in another part of this Journal, an account of the mode of preparing that in general use.—Edit.

